# 4-(4-Bromo­phen­yl)-5-oxo-1,2,3,4,5,6,7,8-octa­hydro­quinazoline-2-thione

**DOI:** 10.1107/S1600536809028761

**Published:** 2009-07-25

**Authors:** Min Xie, Changquan Deng, Jie Zheng, Yulin Zhu

**Affiliations:** a School of Chemistry and Environment, South China Normal University, Guangzhou 510006, People’s Republic of China

## Abstract

The title compound, C_14_H_13_BrN_2_OS, was synthesized from the multicomponent reaction between thio­urea, 4-bromo­benzaldehyde and cyclo­hexane-1,3-dione. The crystal packing is stabilized by inter­molecular N—H⋯O, N—H⋯S, C—H⋯O and C—H⋯S hydrogen bonds. Br⋯O inter­actions [3.183 (3) Å] are also observed in the crystal structure.

## Related literature

For the pharmaceutical applications of 4-aryl-5-oxo-1,2,3,4,5,6,7,8-octa­hydro­quinazoline-2-thio­nes, see: Kappe & Stadler (2004 ▶); Sarac *et al.* (1997 ▶, 1999 ▶); Yarima *et al.*, (2003 ▶). For background information on halogen bonding, see: Damodharana *et al.* (2004 ▶); Sureshan *et al.* (2001 ▶); Yang *et al.* (2008 ▶).
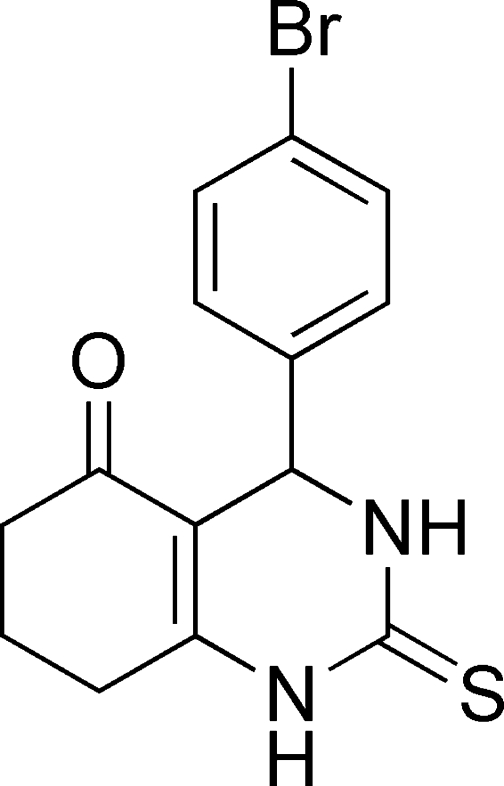


## Experimental

### 

#### Crystal data

C_14_H_13_BrN_2_OS
*M* *_r_* = 337.23
Triclinic, 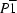

*a* = 7.0395 (11) Å
*b* = 8.1859 (13) Å
*c* = 13.286 (2) Å
α = 105.329 (2)°
β = 91.279 (2)°
γ = 103.854 (2)°
*V* = 713.9 (2) Å^3^
*Z* = 2
Mo *K*α radiation
μ = 3.02 mm^−1^
*T* = 293 K
0.25 × 0.25 × 0.20 mm

#### Data collection

Bruker APEXII area-detector diffractometer
Absorption correction: multi-scan (*SADABS*; Bruker, 2004 ▶) *T*_min_ = 0.429, *T*_max_ = 0.547
3953 measured reflections
2744 independent reflections
1880 reflections with *I* > 2σ(*I*)
*R*_int_ = 0.021

#### Refinement

*R*[*F* ^2^ > 2σ(*F* ^2^)] = 0.046
*wR*(*F* ^2^) = 0.117
*S* = 1.04
2744 reflections
174 parameters
H-atom parameters constrained
Δρ_max_ = 0.42 e Å^−3^
Δρ_min_ = −0.60 e Å^−3^

### 

Data collection: *APEX2* (Bruker, 2004 ▶); cell refinement: *SAINT* (Bruker, 2004 ▶); data reduction: *SAINT*; program(s) used to solve structure: *SHELXS97* (Sheldrick, 2008 ▶); program(s) used to refine structure: *SHELXL97* (Sheldrick, 2008 ▶); molecular graphics: *SHELXTL* (Sheldrick, 2008 ▶); software used to prepare material for publication: *SHELXTL*.

## Supplementary Material

Crystal structure: contains datablocks global, I. DOI: 10.1107/S1600536809028761/zl2225sup1.cif

Structure factors: contains datablocks I. DOI: 10.1107/S1600536809028761/zl2225Isup2.hkl

Additional supplementary materials:  crystallographic information; 3D view; checkCIF report

## Figures and Tables

**Table 1:** Hydrogen-bond geometry (Å, °)

*D*—H⋯*A*	*D*—H	H⋯*A*	*D*⋯*A*	*D*—H⋯*A*
C2—H2*A*⋯S1^i^	0.97	2.98	3.781 (4)	140
N2—H2⋯S1^ii^	0.86	2.55	3.380 (3)	161
N1—H1⋯O1^iii^	0.86	2.00	2.832 (4)	164
C4—H4*A*⋯O1^iii^	0.97	2.59	3.361 (4)	137

Symmetry codes: (i) 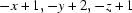; (ii) 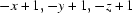; (iii) 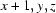.

**Fig. 1.**
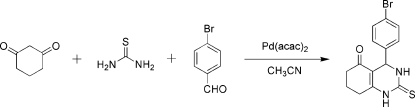
Palladium(II) 2,4-pentanedionate catalyzed synthesis of the title compound.

**Fig. 2.**
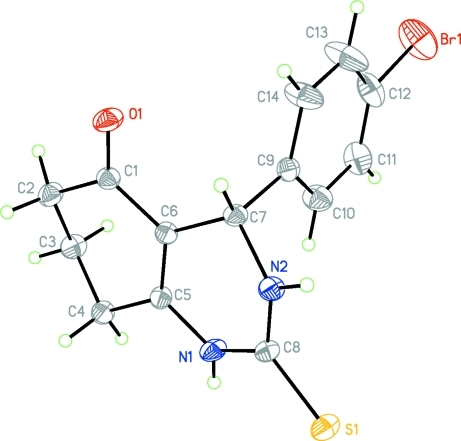
View of the title compound showing the atom-labelling scheme. Ellipsoids are drawn at the 50% probability level.

**Fig. 3.**
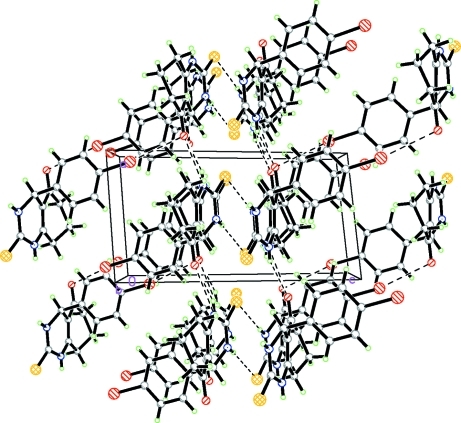
Perspective view of the packing of the title compound. Dashed lines stand for N1—H1···O1 and N2—H2···S1 intermolecular hydrogen bonds and Br1···O1 interactions.

## supplementary crystallographic information

### Comment

4-Aryl-5-oxo-1,2,3,4,5,6,7,8-octahydroquinazoline-2-thiones have received much attention recently because of their pharmaceutical applications. (Kappe & Stadler, 2004; Sarac *et al.*, 1997; Sarac *et al.*, 1999). For example, the calcium antagonist activity of the compounds was tested in vitro on isolated rat ileum and lamb carotid artery. (Yarima *et al.*, 2003). As part of our on going studies on the synthesis of quinazolinethiones, the title compound was isolated under Biginelli reaction conditions (Figure 1).

The reaction between thiourea, 4-bromobenzaldehyde, and 1,3-cyclohexanedione instead of an open-chain dicarbonyl compound in the presence of palladium(II) 2,4-pentanedionate as catalyst proceeded to give the title compound in excellent yield. A representation of the title compound is given in Figure 2. There are no unusual bond lengths and angles in the compound. The molecules in the structure are linked via N1—H1···O1 and paired N2—H2···S1 intermolecular hydrogen bonds. The bromine atom Br1 exhibits a Br···O halogen bond with oxygen atom O1 (Figure 3). (Damodharana *et al.*, 2004; Sureshan *et al.*, 2001; Yang *et al.*, 2008). The Br1···O1 distance of this interaction is 3.182 Å, which is less than the sum of their van der Waals radii.

### Experimental

A mixture of thiourea (0.91 g, 12 mmol), 4-bromobenzaldehyde (1.84 g, 10 mmol), 1,3-cyclohexanedione (1.12 g, 10 mmol), and palladium(II) 2,4-pentanedionate (0.0020 mg) was refluxed in acetonitrile (12 ml) at 353 K for 4 h. After being cooled to room temperature, the reaction mixture was poured into water. The white precipitate was filtered off with a silica pad, washed twice with water, and the filtrate was then dried under vacuum to yield the product. Single crystals of the title compound were obtained by slow evaporation from ethanol at room temperature to yield colourless, block-shaped crystal.

### Refinement

The H atoms were positioned geometrically and allowed to ride on their parent atoms, with C—H = 0.93–0.97 Å and N—H = 0.86 Å, respectively, and *U*_iso_ = 1.2_eq_(parent atom).

### Crystal data

**Table:**

C_14_H_13_BrN_2_OS	*Z* = 2
*M**_r_* = 337.23	*F*(000) = 340
Triclinic, *P*1	*D*_x_ = 1.569 Mg m^−^^3^
Hall symbol: -P 1	Mo *K*α radiation, λ = 0.71073 Å
*a* = 7.0395 (11) Å	Cell parameters from 1089 reflections
*b* = 8.1859 (13) Å	θ = 2.7–23.6°
*c* = 13.286 (2) Å	µ = 3.02 mm^−^^1^
α = 105.329 (2)°	*T* = 293 K
β = 91.279 (2)°	Block, colourless
γ = 103.854 (2)°	0.25 × 0.25 × 0.20 mm
*V* = 713.9 (2) Å^3^	

### Data collection

**Table:**

Bruker APEXII area-detector diffractometer	2744 independent reflections
Radiation source: fine-focus sealed tube	1880 reflections with *I* > 2σ(*I*)
graphite	*R*_int_ = 0.021
φ and ω scans	θ_max_ = 26.0°, θ_min_ = 1.6°
Absorption correction: multi-scan (*APEX2*; Bruker, 2004)	*h* = −8→8
*T*_min_ = 0.429, *T*_max_ = 0.547	*k* = −10→10
3953 measured reflections	*l* = −16→16

### Refinement

**Table:**

Refinement on *F*^2^	Secondary atom site location: difference Fourier map
Least-squares matrix: full	Hydrogen site location: inferred from neighbouring sites
*R*[*F*^2^ > 2σ(*F*^2^)] = 0.046	H-atom parameters constrained
*wR*(*F*^2^) = 0.117	*w* = 1/[σ^2^(*F*_o_^2^) + (0.0382*P*)^2^ + 0.8552*P*] where *P* = (*F*_o_^2^ + 2*F*_c_^2^)/3
*S* = 1.04	(Δ/σ)_max_ < 0.001
2744 reflections	Δρ_max_ = 0.42 e Å^−^^3^
174 parameters	Δρ_min_ = −0.60 e Å^−^^3^
0 restraints	Extinction correction: *SHELXL97* (Sheldrick, 2008), Fc^*^=kFc[1+0.001xFc^2^λ^3^/sin(2θ)]^-1/4^
Primary atom site location: structure-invariant direct methods	Extinction coefficient: 0.0058 (17)

### Special details

**Table:**

Geometry. All esds (except the esd in the dihedral angle between two l.s. planes) are estimated using the full covariance matrix. The cell esds are taken into account individually in the estimation of esds in distances, angles and torsion angles; correlations between esds in cell parameters are only used when they are defined by crystal symmetry. An approximate (isotropic) treatment of cell esds is used for estimating esds involving l.s. planes.
Refinement. Refinement of F^2^ against ALL reflections. The weighted R-factor wR and goodness of fit S are based on F^2^, conventional R-factors R are based on F, with F set to zero for negative F^2^. The threshold expression of F^2^ > 2sigma(F^2^) is used only for calculating R-factors(gt) etc. and is not relevant to the choice of reflections for refinement. R-factors based on F^2^ are statistically about twice as large as those based on F, and R- factors based on ALL data will be even larger.

### Fractional atomic coordinates and isotropic or equivalent isotropic displacement parameters (Å^2^)

**Table:**

	*x*	*y*	*z*	*U*_iso_*/*U*_eq_	
Br1	0.08077 (11)	0.17375 (8)	−0.08352 (4)	0.0927 (3)	
S1	0.81565 (14)	0.62120 (13)	0.47136 (8)	0.0395 (3)	
N2	0.4706 (4)	0.6637 (4)	0.4168 (2)	0.0322 (7)	
H2	0.4223	0.6000	0.4568	0.039*	
C8	0.6646 (5)	0.7134 (5)	0.4188 (3)	0.0294 (8)	
C7	0.3316 (5)	0.7096 (4)	0.3511 (3)	0.0291 (8)	
H7	0.2139	0.7176	0.3881	0.035*	
C6	0.4253 (5)	0.8861 (4)	0.3369 (3)	0.0282 (8)	
C5	0.6241 (5)	0.9449 (4)	0.3455 (3)	0.0287 (8)	
C1	0.3007 (5)	0.9915 (4)	0.3130 (3)	0.0303 (8)	
C4	0.7274 (5)	1.1109 (5)	0.3220 (3)	0.0352 (9)	
H4A	0.8475	1.0964	0.2906	0.042*	
H4B	0.7626	1.2047	0.3867	0.042*	
C2	0.3965 (5)	1.1566 (5)	0.2867 (3)	0.0396 (9)	
H2A	0.4084	1.2547	0.3484	0.047*	
H2B	0.3129	1.1722	0.2328	0.047*	
C3	0.5987 (5)	1.1589 (5)	0.2486 (3)	0.0402 (9)	
H3A	0.5851	1.0768	0.1796	0.048*	
H3B	0.6606	1.2749	0.2426	0.048*	
N1	0.7383 (4)	0.8485 (4)	0.3772 (2)	0.0350 (7)	
H1	0.8622	0.8751	0.3703	0.042*	
C9	0.2714 (5)	0.5708 (4)	0.2475 (3)	0.0321 (8)	
C12	0.1586 (8)	0.3298 (5)	0.0523 (3)	0.0557 (12)	
C14	0.0763 (6)	0.4943 (6)	0.2134 (4)	0.0598 (13)	
H14	−0.0197	0.5240	0.2564	0.072*	
C10	0.4095 (6)	0.5213 (6)	0.1814 (3)	0.0482 (11)	
H10	0.5424	0.5704	0.2027	0.058*	
C11	0.3538 (7)	0.4003 (6)	0.0842 (3)	0.0545 (12)	
H11	0.4483	0.3674	0.0411	0.065*	
C13	0.0205 (8)	0.3738 (7)	0.1163 (4)	0.0802 (18)	
H13	−0.1120	0.3228	0.0947	0.096*	
O1	0.1229 (4)	0.9472 (3)	0.3167 (2)	0.0426 (7)	

### Atomic displacement parameters (Å^2^)

**Table:**

	*U*^11^	*U*^22^	*U*^33^	*U*^12^	*U*^13^	*U*^23^
Br1	0.1179 (6)	0.0830 (5)	0.0453 (3)	−0.0032 (4)	0.0010 (3)	−0.0098 (3)
S1	0.0319 (6)	0.0473 (6)	0.0477 (6)	0.0135 (4)	0.0060 (4)	0.0239 (5)
N2	0.0243 (17)	0.0368 (17)	0.0377 (17)	0.0038 (13)	0.0046 (13)	0.0172 (14)
C8	0.027 (2)	0.036 (2)	0.0277 (18)	0.0093 (16)	0.0019 (14)	0.0123 (15)
C7	0.0207 (18)	0.0322 (19)	0.0358 (19)	0.0052 (15)	0.0042 (15)	0.0127 (15)
C6	0.0252 (19)	0.0285 (18)	0.0306 (18)	0.0044 (15)	0.0049 (14)	0.0099 (14)
C5	0.0245 (19)	0.0284 (18)	0.0348 (19)	0.0065 (15)	0.0069 (15)	0.0114 (15)
C1	0.026 (2)	0.0326 (19)	0.0319 (19)	0.0073 (15)	0.0046 (15)	0.0079 (15)
C4	0.026 (2)	0.031 (2)	0.050 (2)	0.0026 (16)	0.0055 (17)	0.0165 (17)
C2	0.030 (2)	0.039 (2)	0.053 (2)	0.0100 (17)	0.0058 (18)	0.0179 (18)
C3	0.034 (2)	0.041 (2)	0.051 (2)	0.0052 (18)	0.0070 (18)	0.0251 (19)
N1	0.0208 (16)	0.0410 (18)	0.0496 (19)	0.0077 (13)	0.0072 (14)	0.0231 (15)
C9	0.030 (2)	0.0297 (19)	0.038 (2)	0.0042 (16)	0.0037 (16)	0.0161 (16)
C12	0.073 (3)	0.040 (2)	0.041 (2)	−0.003 (2)	0.007 (2)	0.0044 (19)
C14	0.034 (2)	0.074 (3)	0.050 (3)	−0.002 (2)	0.006 (2)	−0.003 (2)
C10	0.037 (2)	0.054 (3)	0.049 (3)	0.010 (2)	0.0076 (19)	0.008 (2)
C11	0.067 (3)	0.051 (3)	0.044 (3)	0.014 (2)	0.015 (2)	0.010 (2)
C13	0.045 (3)	0.092 (4)	0.064 (3)	−0.013 (3)	0.000 (3)	−0.017 (3)
O1	0.0251 (15)	0.0507 (17)	0.0573 (18)	0.0117 (12)	0.0069 (12)	0.0221 (14)

### Geometric parameters (Å, °)

**Table:**

Br1—C12	1.895 (4)	C4—H4B	0.9700
Br1—O1^i^	3.183 (3)	C2—C3	1.519 (5)
S1—C8	1.678 (4)	C2—H2A	0.9700
N2—C8	1.326 (4)	C2—H2B	0.9700
N2—C7	1.474 (4)	C3—H3A	0.9700
N2—H2	0.8600	C3—H3B	0.9700
C8—N1	1.364 (4)	N1—H1	0.8600
C7—C6	1.500 (5)	C9—C14	1.376 (5)
C7—C9	1.510 (5)	C9—C10	1.386 (5)
C7—H7	0.9800	C12—C13	1.356 (6)
C6—C5	1.358 (5)	C12—C11	1.366 (7)
C6—C1	1.453 (5)	C14—C13	1.383 (6)
C5—N1	1.382 (4)	C14—H14	0.9300
C5—C4	1.495 (5)	C10—C11	1.384 (6)
C1—O1	1.222 (4)	C10—H10	0.9300
C1—C2	1.492 (5)	C11—H11	0.9300
C4—C3	1.504 (5)	C13—H13	0.9300
C4—H4A	0.9700		
			
C12—Br1—O1^i^	154.81 (16)	C1—C2—H2B	108.9
C8—N2—C7	124.8 (3)	C3—C2—H2B	108.9
C8—N2—H2	117.6	H2A—C2—H2B	107.7
C7—N2—H2	117.6	C4—C3—C2	111.6 (3)
N2—C8—N1	116.3 (3)	C4—C3—H3A	109.3
N2—C8—S1	123.1 (3)	C2—C3—H3A	109.3
N1—C8—S1	120.6 (3)	C4—C3—H3B	109.3
N2—C7—C6	108.5 (3)	C2—C3—H3B	109.3
N2—C7—C9	111.0 (3)	H3A—C3—H3B	108.0
C6—C7—C9	112.0 (3)	C8—N1—C5	123.3 (3)
N2—C7—H7	108.4	C8—N1—H1	118.3
C6—C7—H7	108.4	C5—N1—H1	118.3
C9—C7—H7	108.4	C14—C9—C10	117.5 (4)
C5—C6—C1	120.8 (3)	C14—C9—C7	121.0 (3)
C5—C6—C7	120.1 (3)	C10—C9—C7	121.4 (3)
C1—C6—C7	119.1 (3)	C13—C12—C11	120.5 (4)
C6—C5—N1	119.4 (3)	C13—C12—Br1	119.9 (4)
C6—C5—C4	122.9 (3)	C11—C12—Br1	119.6 (3)
N1—C5—C4	117.7 (3)	C9—C14—C13	121.1 (4)
O1—C1—C6	120.6 (3)	C9—C14—H14	119.4
O1—C1—C2	121.3 (3)	C13—C14—H14	119.4
C6—C1—C2	118.1 (3)	C11—C10—C9	121.4 (4)
C5—C4—C3	110.8 (3)	C11—C10—H10	119.3
C5—C4—H4A	109.5	C9—C10—H10	119.3
C3—C4—H4A	109.5	C12—C11—C10	119.3 (4)
C5—C4—H4B	109.5	C12—C11—H11	120.3
C3—C4—H4B	109.5	C10—C11—H11	120.3
H4A—C4—H4B	108.1	C12—C13—C14	120.2 (5)
C1—C2—C3	113.4 (3)	C12—C13—H13	119.9
C1—C2—H2A	108.9	C14—C13—H13	119.9
C3—C2—H2A	108.9		
			
C7—N2—C8—N1	16.5 (5)	C1—C2—C3—C4	−50.8 (5)
C7—N2—C8—S1	−164.9 (3)	N2—C8—N1—C5	7.6 (5)
C8—N2—C7—C6	−31.3 (4)	S1—C8—N1—C5	−171.1 (3)
C8—N2—C7—C9	92.1 (4)	C6—C5—N1—C8	−12.3 (5)
N2—C7—C6—C5	24.8 (4)	C4—C5—N1—C8	167.8 (3)
C9—C7—C6—C5	−98.0 (4)	N2—C7—C9—C14	126.7 (4)
N2—C7—C6—C1	−155.8 (3)	C6—C7—C9—C14	−111.9 (4)
C9—C7—C6—C1	81.3 (4)	N2—C7—C9—C10	−56.1 (4)
C1—C6—C5—N1	174.5 (3)	C6—C7—C9—C10	65.3 (4)
C7—C6—C5—N1	−6.1 (5)	O1^i^—Br1—C12—C13	63.0 (6)
C1—C6—C5—C4	−5.6 (5)	O1^i^—Br1—C12—C11	−115.5 (5)
C7—C6—C5—C4	173.8 (3)	C10—C9—C14—C13	−0.7 (7)
C5—C6—C1—O1	−171.9 (3)	C7—C9—C14—C13	176.6 (5)
C7—C6—C1—O1	8.8 (5)	C14—C9—C10—C11	0.5 (6)
C5—C6—C1—C2	6.5 (5)	C7—C9—C10—C11	−176.8 (4)
C7—C6—C1—C2	−172.9 (3)	C13—C12—C11—C10	−1.9 (7)
C6—C5—C4—C3	−23.7 (5)	Br1—C12—C11—C10	176.6 (3)
N1—C5—C4—C3	156.2 (3)	C9—C10—C11—C12	0.8 (7)
O1—C1—C2—C3	−159.6 (4)	C11—C12—C13—C14	1.8 (9)
C6—C1—C2—C3	22.0 (5)	Br1—C12—C13—C14	−176.8 (4)
C5—C4—C3—C2	50.7 (4)	C9—C14—C13—C12	−0.4 (9)

Symmetry codes: (i) −*x*, −*y*+1, −*z*.

### Hydrogen-bond geometry (Å, °)

**Table:**

*D*—H···*A*	*D*—H	H···*A*	*D*···*A*	*D*—H···*A*
C2—H2A···S1^ii^	0.97	2.98	3.781 (4)	140
N2—H2···S1^iii^	0.86	2.55	3.380 (3)	161
N1—H1···O1^iv^	0.86	2.00	2.832 (4)	164
C4—H4A···O1^iv^	0.97	2.59	3.361 (4)	137

Symmetry codes: (ii) −*x*+1, −*y*+2, −*z*+1; (iii) −*x*+1, −*y*+1, −*z*+1; (iv) *x*+1, *y*, *z*.
